# Synergistic Interaction of Piperine and Thymol on Attenuation of the Biofilm Formation, Hyphal Morphogenesis and Phenotypic Switching in *Candida albicans*

**DOI:** 10.3389/fcimb.2021.780545

**Published:** 2022-01-19

**Authors:** Arumugam Priya, Srinivasan Nivetha, Shunmugiah Karutha Pandian

**Affiliations:** Department of Biotechnology, Alagappa University, Karaikudi, India

**Keywords:** *C. albicans*, synergism, antibiofilm, combinatorial therapy, piperine, thymol, antihyphae

## Abstract

The incidence of fungal infections has significantly increased in recent years due to the emergence of antifungal resistance. Biofilm formation is considered to be a major contributor to both the infectious diseases and to antimicrobial resistance. Consequently, biofilm-associated infections are often problematic to treat with existing therapeutics. Adhesion of *C. albicans* to the host surface or implanted materials followed by hyphal invasion and biofilm formation enhances *C. albicans* pathogenicity and virulence. Thus, developing a therapeutic agent that inhibits candidal adherence, biofilm development and morphological switching could improve clinical management of infections. The present investigation studied two emerging and alternatives strategies, namely antibiofilm and combinatorial approach, to attenuate biofilm formation and the expression of *Candida* virulence factors. Piperine and thymol are major bioactive components of pepper and thyme, respectively. These phytochemicals are known to possess numerous biological activities, including recently reported antibiofilm effects against *C. albicans*. The minimum biofilm inhibitory concentration (MBIC) of both phytochemicals was determined to be 32 µg/ml. The phytochemical treatment of *Candida* biofilms using piperine and thymol revealed synergistic effects at four different combinations of concentrations, i.e. 8 and 8, 8 and 4, 8 and 2 and 4 and 8 µg/ml. These synergistic combinations resulted in the significant reduction in adherence of *Candida*, hyphal extension and morphological transformation. Moreover, limited exposure of synergistic combinations controlled the hyphal elongation. Results were validated through the gene expression analysis. Results from the present investigation suggest that piperine and thymol can be synergistically employed for the treatment of biofilm-associated *C. albicans* infection.

## Introduction

The genus *Candida* is ubiquitously distributed and comprises more than 350 species of “yeast-like fungi” (Williams and Lewis, 2011; Williams et al., 2012). The majority of the *Candida* species are unable to grow at 37°C and thus not generally associated with colonization of humans (Schauer and Hanschke, 1999). Nevertheless, certain species such as *Candida albicans*, *C. glabrata*, *C. tropicalis*, *C. krusei*, *C. parapsilosis* and *C. dubliniensis* exist as a part of the human commensal microbiota that can transmute to opportunistic pathogens under favourable conditions (Fisher et al., 1987; Vanden Abbeele et al., 2008; Luque et al., 2009; Martins et al., 2010; Meurman et al., 2011). These yeast reside in an individual’s oral cavity, gastrointestinal tract and vagina (Shao et al., 2007). Infections caused by *Candida* species are generally known as candidiasis, and *Candida* infections associated with the oral cavity are described as oral candidiasis. Of the *Candida* species isolated from humans, *C. albicans* is the most predominant species associated with both health and disease (LaFleur et al., 2010; de Oliveira Mima et al., 2010). Several mycological studies have shown that *C. albicans* represents more than 80% of isolates in all forms of candidiasis (Pfaller and Diekema, 2007). Of the various mucosal sites, colonization of *C. albicans* in the oral cavity is more significant, with 80% of healthy individuals carrying *C. albicans* in their mouth (Williams et al., 2012). Predisposing risk factors associated with oral candidiasis include decreased salivary production, immunocompromised status, smoking, diabetes, malnutrition, impaired oral flora and/or poor oral hygiene (Rautemaa and Ramage, 2011). The local breach in the mucosal surfaces caused by mucositis, radiation and trauma are also considered as predisposing factors. Colonization of *Candida* in the oral cavity may occur at several sites including biotic surfaces such as tongue, buccal mucosa, palate and abiotic surfaces such as denture (Nett et al., 2010). Denture stomatitis is considered one of the most prevalent forms of oral candidiasis, affecting up to 70% of denture wearers (Wilson, 1998; Senpuku et al., 2003). *C. albicans* can directly interact either with the denture acrylic surface or with plaque-forming bacteria (Chandra et al., 2001).

Pathogenesis and virulence of *C. albicans* rely on numerous factors which altogether support the organism to survive and proliferate in the healthy host surpassing the immune factors and attack of antibiotics (Nett et al., 2010). Among the various virulence factors, most imperative are those that support adherence to the host tissues and implanted devices, biofilm formation and secretion of hydrolytic enzymes such as phospholipase, lipase, proteases and haemolysins (Sardi et al., 2013). Additionally, the invasiveness of *C. albicans* is contributed by the proficiency for phenotypic switching between yeast and filamentous forms (Cullen and Sprague, 2012).

Biofilm formation by fungal species is defined as a major standard for pathogenesis (Martinez and Fries, 2010). Studies suggest that majority of the infections caused by *C. albicans* are associated with biofilm formation. Early biofilm formation initiates when *C. albicans* instigates an interaction with the host tissue or with the implant surfaces. Increased pathogenicity is observed in the biofilm form of infection as the biofilm matrix defends *Candida* cells from the host immunological responses and from antifungal therapy (Ramage et al., 2004). Biofilm-mediated drug resistance is emerging as a major contributor to numerous human ailments. *C. albicans* cells encased within the biofilm matrix are less susceptible to the antifungals compared to their planktonic counterparts (Kuhn and Ghannoum, 2004; Uppuluri et al., 2009; Rautemaa and Ramage, 2011). Numerous broad-spectrum antifungal drugs are available currently for the treatment of *Candida* infections, which include azole drugs (fluconazole, itraconazole, clotrimazole and ketoconazole), echinocandins (micafungin, caspofungin) and polyenes (amphotericin B). Adverse side effects and rising resistance development have made the usage of antifungals sceptical. Thus, research on the identification of therapeutic regimens has shifted the focus in targeting the biofilm and other virulence factors of the pathogen rather than affecting the survival (Bandara et al., 2017; Vila et al., 2017). With the traditional practice of medicinal plants for the cure of numerous ailments, studies have demonstrated the usage of plant-based active molecules to overcome biofilm-associated *Candida* infections (Priya and Pandian, 2020; Muthamil et al., 2020). Numerous bioactives with antibiofilm activity have been identified from various natural sources (Abraham et al., 2012). Yet, most of the bioactives fail to cross clinical trials due to high concentration and/or toxic effects. To overcome this, a recent approach, combinatorial drug therapy, has gained more attention due to the benefits of reduced toxicity, improved efficacy and being devoid of antibiotic resistance with increased potential than individual drug candidates (Chanda and Rakholiya, 2011).

With this backdrop, the present study aimed to evaluate the synergistic antivirulence potential of two phytochemicals, piperine and thymol, against *C. albicans*. Our earlier study has reported the antibiofilm potential of piperine, a major bioactive component of the pepper seeds against *C. albicans* (Priya and Pandian, 2020). Thymol is a major constituent in the essential oil of thyme plant (*Thymus vulgaris*) and was previously reported to possess antibiofilm and hyphal inhibitory activity against *C. albicans* (Braga et al., 2007; Braga et al., 2008). The present work is expected to pave way for the prevention of biofilm-associated *C. albicans* infection (antibiofilm activity) and invasive candidiasis (antihyphal activity).

## Materials and Methods

### Ethical Statement

In the present study, human blood and buccal epithelial cells were collected from healthy individuals after procuring a written informed consent. The experimental protocol and the use of human blood and HBECs were assessed and approved by the Institutional Ethical Committee, Alagappa University, Karaikudi (IEC Ref No. IEC/AU/2018/5). All the methods were carried out in accordance with relevant guidelines.

## Fungal Strain and Growth Conditions

Reference fungal strain *C. albicans* 90028 from the American Type Culture Collection (ATCC) was used in the present study. In addition to the reference strain, four different clinical isolates of *C. albicans*, namely CI-1 (GenBank accession no.: MF445114.1); CI-2 (GenBank accession no.: MF445113.1); CI-3 (GenBank accession no.: MF445115.1) and CI-4 (GenBank accession no.: MF445116.1), were included to evaluate the efficacy of phytocompounds and their combinatorial effects. Culture was maintained in YEPD (1% yeast extract, 2% peptone and 2% dextrose) agar plates at 4°C. Routine culturing was carried out in YEPD broth and incubated at 37°C with constant shaking at 160 rpm. Assays associated with biofilm formation and hyphal development were performed with spider medium (1% of mannitol, 0.2% of dipotassium hydrogen phosphate and 1% of nutrient broth). Hyphal induction for hyphal to yeast transition assay was carried out using RPMI medium (HiMedia, India).

### Phytochemicals

Piperine (PubChem CID: 638024) and thymol (PubChem CID: l6989) were commercially procured from HiMedia and Alfa Aesar, respectively. Stock solutions of both the phytocompounds were prepared with the concentration of 50 mg/ml in methanol. The highest volume of compound used in the test group was taken as the volume of methanol for vehicle control in each assay.

### Influence of Piperine and Thymol on Viability and Biofilm Formation of *C. albicans*

The impact of individual effects of piperine and thymol on the growth of *C. albicans* reference and clinical strains was determined by microbroth dilution assay as per the CLSI guideline (Wayne, 2008). The determination of the biofilm inhibitory concentration was performed as described earlier (Priya and Pandian, 2020). Briefly, in a 24-well microtiter plate (MTP), 1 ml of spider broth was added with increasing concentrations of individual phytochemicals in the range of 2–1,024 µg/ml. The medium added with methanol served as vehicle control. To this, 1% of overnight culture of *C. albicans* was inoculated. Wells containing medium alone served as blank. The plates were incubated at 37°C for 48 h at static conditions. Following incubation, the planktonic part was discarded and the loosely bound biofilm cells were detached by carefully rinsing the wells with sterile PBS. Plates were air dried. Biofilm cells bound to the surface of the MTP were stained with 0.4% crystal violet and incubated at room temperature for 5 min, and the excess unbound stain was decanted. Plates were then destained with 10% glacial acetic acid solution and read at 595 nm in a multifunctional spectrophotometer. Inhibition of biofilm formation was calculated in percentage using the following formula:


$$\%\;of\;biofilm\;inhibition=\frac{{\text{(Control OD}}_{570}{\text{ nm - Treated OD}}_{570}\;nm)}{{\text{Control OD}}_{570}\text{ nm}}$$

MBIC was considered as the minimum concentration required to inhibit a minimum of 85% or more of biofilm when compared to the control biofilm.

### Checkerboard Assay to Identify the Combinatorial Efficacy of Piperine and Thymol Against *C. albicans* Biofilm

The interaction between two phytochemicals piperine and thymol in exhibiting increased antibiofilm activity at lesser concentrations than their individual effect was evaluated through two-dimensional checkerboard assay for *C. albicans* reference and clinical strains. A total of 25 different antibiofilm combinations were analysed between phytochemicals in a decreasing concentration ranging from biofilm inhibitory concentration (BIC) to 1/16 BIC. The assay was performed in a 48-well MTP. In the first column, piperine was added to the spider medium at the BIC concentration, and then 2-fold dilutions were made for the subsequent plate columns. In a similar way, BIC of thymol was added to the first row and 2-fold dilutions were made for the subsequent plate rows. The last drug concentration in the row and column of the plate was set to 0 µg/ml, to examine the activity of individual phytochemicals at various test concentrations in combination. Appropriate control, vehicle control and negative controls were maintained in parallel. Plates were incubated at 37°C for 48 h in static conditions. At the end of the incubation period, the plates were processed to analyse the antibiofilm activity with the similar procedure detailed in the previous methods section. The percentage of biofilm inhibition by individual and combinations of piperine and thymol was calculated, and a heatmap was generated. The fractional inhibitory concentration (FIC) was calculated individually, and the FIC index (FICI) was calculated by summing up the FIC of both the phytocompounds as the following:


$$\text{FICI}={\text{FIC}}_{\text{A}}+{\text{FIC}}_{\text{B}}$$

FIC_A_ =BIC of A in combination with B/BIC of A alone

FIC_B_ =BIC of B in combination with A/BIC of B alone

The combinations were said to be synergistic when the FICI is ≤ 0.5. Combinations with the FICI in the range of greater than 0.5 to less than or equal to 4 is said to be indifferent. When FICI is greater than 4, the phytochemicals in the combination were known to exhibit antagonistic effect.

### Effect of Combinations of Piperine and Thymol on Viability of *C. albicans*

Checkerboard assay was performed with YEPD broth and incubated at 37°C for 24 h. Followed by incubation, culture was mixed well and 5 µl from each well was spotted on the agar plate which was incubated for 37°C for 24 h. Similarly, *C. albicans* grown in the absence and presence of the identified synergistic combinations were grown and CFU analysis was performed.

### Analysis of Biofilm Cell Count Under the Influence of Individual and Combined Activity of Piperine and Thymol

*C. albicans* was allowed to form biofilm in the absence and presence of piperine, thymol and their synergistic combinations in spider medium for 48 h at 37°C. Planktonic cells were removed, and loosely bound cells were removed by gentle wash with PBS. The adhered biofilm cells were resuspended in PBS. Serial dilutions were made and plated on YEPD agar plates. CFU/mL was calculated after incubation for 24 h at 37°C, and log reduction in biofilm cells compared to control was plotted as graph.

### Microscopic Visualization of Biofilm Architecture

Biofilm architecture and filamentous morphology of *C. albicans* in the absence and presence of individual and synergistic combinations of piperine and thymol were microscopically evaluated. Briefly, *C. albicans* was allowed to form biofilm on a 1 × 1-cm glass surface for 48 h at 37°C. After incubation, the glass slides were carefully removed and processed as mentioned earlier. Crystal violet-stained glass slides were observed under the light microscope (Nikon Eclipse 80i, USA) at ×400 magnification.

### Checkerboard Assay to Evaluate the Combinatorial Efficacy of Piperine and Thymol Against *C. albicans* Hyphae

Checkerboard assay was performed as mentioned previously. Instead of liquid medium, solid spider agar was prepared and 5% foetal bovine serum was supplemented after the agar reached ambient temperature to induce the filamentation. To this mixture, various concentrations of piperine and thymol were added, mixed well and poured on glass plates. After proper solidification, 5 µl of *C. albicans* overnight culture was spotted on the centre of the medium. Plates were incubated at 37°C for 5–7 days. Variation in the filamentous morphology was observed and documented using the gel documentation system (Bio-Rad Gel Doc™ XR^+^).

### Influence of Individual and Synergistic Combinations of Piperine and Thymol on *C. albicans* Phenotypic Switching

The ability of the phytochemicals to control *C. albicans* phenotypic switching between yeast to hyphal and vice versa was appraised by the following methods.

#### (A) Yeast to Hyphal Morphogenesis

*C. albicans* was grown on YEPD medium supplemented with 10% FBS to facilitate hyphal induction. Individual and synergistic combinations of piperine and thymol were added separately and incubated at 37°C with constant shaking at 160 rpm for 4 h. Further, the transition in the morphology was observed under the microscope (Nikon Eclipse Ts2R, Japan).

#### (B) Hyphal to Yeast Transition

Reversal of hyphal to yeast form under the influence of individual and synergistic combinations of piperine and thymol was performed in hyphal-inducing conditions. Briefly, the cells were inoculated into the RPMI medium and allowed to develop hyphae for 4 h at 37°C. After ensuring the presence of hyphal forms, phytochemicals at their individual and synergistic combination concentrations were added and further incubated for 2 h. Subsequently, the morphology was microscopically evaluated.

### Post Antihyphal Effect

*C. albicans* cells were briefly exposed to the phytochemicals for 1 h at 37°C with gentle shaking. After incubation, the compounds were removed by three rounds of centrifugation. Subsequently, phytochemical-exposed *C. albicans* cells were used as inoculum and introduced into RPMI medium which was further incubated for 2 h. Later, cells were observed under the microscope.

### Antifungal Sensitivity Assay

Sensitivity of the *C. albicans* cells treated with individual and synergistic combinations of piperine and thymol to antifungals such as miconazole, itraconazole, ketoconazole and nystatin was evaluated by disc diffusion assay. Briefly, *C. albicans* cells were grown in the absence and presence of individual and combination of phytochemicals overnight in YEPD medium at 37°C. The cultures were inoculated on the YEPD agar plates, and antifungal discs were placed on the centre and incubated for 24 h at 37°C. The zone of clearance was noted.

### Gene Expression Analysis

The dynamics in the expression of candidate genes responsible for biofilm formation, hyphal regulation, adhesion between the control and synergistic combinations of piperine and thymol-treated *C. albicans* was assessed through qPCR analysis. RNA from control and phytocompound-treated *C. albicans* was extracted by the TRIzol method using commercial TRI reagent (Sigma-Aldrich, India). Extracted RNA was converted to cDNA using the high-capacity cDNA Reverse Transcription Kit (Applied Biosystems, United States), and qPCR analysis was performed with SYBR Green Master Mix (Applied Biosystems, United States) with the Applied Biosystems^®^ 7500 Real-Time PCR system. A list of candidate genes, primer details and functions is provided in **Table 1**. Alterations in the gene expression were calculated by the ^ΔΔ^CT method.

**Table 1:** Candidate genes, primer details and function in the virulence and pathogenicity of *C. albicans*.

S. no.	Gene	Primer sequence (5′–3′)		Function
		Forward	Reverse	
1	*nrg1*	CCAAGTACCTCCACCAGCAT	GGGAGTTGGCCAGTAAATCA	Negative regulator of filamentous growth. Repress *ece1* and *hwp1*
2	*ume6*	ACCACCACTACCACCACCAC	TATCCCCATTTCCAAGTCCA	Transcriptional activator of filamentous growth. Regulates hyphal elongation and germ tube formation
3	*tup1*	CTTGGAGTTGGCCCATAGAA	TGGTGCCACAATCTGTTGTT	Negative regulator of filamentation. Farnesol-mediated inhibition of filamentation, controls phenotypic switching
4	*efg1*	GCCTCGAGCACTTCCACTGT	TTTTTTCATCTTCCCACATGGTAGT	Regulates switch between white and opaque cells and cell wall dynamics. Essential for biofilm formation and filamentous growth
5	*cph1*	TATGACGCTTCTGGGTTTCC	ATCCCATGGCAATTTGTTGT	Transcription factor involved in pseudohyphal and hyphal formation and phenotypic switching
6	*eap1*	TGTGATGGCGGTTCTTGTTC	GGTAGTGACGGTGATGATAGTGACA	Associated with cell adhesion and filamentation. Instigates adhesion to polystyrene and epithelial cells
7	*ras1*	CCCAACTATTGAGGATTCTTATCGTAAA	TCTCATGGCCAGATATTCTTCTTG	Mediates cell adhesion, induces filamentous growth, regulates phenotypic switching
8	*als1*	CCTATCTGACTAAGACTGCACC	ACAGTTGGATTTGGCAGTGGA	Adhesin. Vital for adhesion to oral mucosa and epithelial cells. Mediates yeast aggregation, a first step in biofilm formation
9	*ece1*	CCAGAAATTGTTGCTCGTGTTGCCA	TCCAGGACGCCATCAAAAACGTTAG	Candidalysin. Regulates biofilm formation and filamentation
10	*hwp1*	GCTCCTGCTCCTGAAATGAC	CTGGAGCAATTGGTGAGGTT	Hyphal wall protein. Essential for hyphal elongation

### Analysis of Cytotoxic Effect of Piperine, Thymol and Their Synergistic Combinations

The toxic effect of phytocompounds and their combinations were analysed on human red blood cells and human buccal epithelial cells (HBECs).

### (A) Haemolytic Activity

Human erythrocytes were collected from healthy volunteers in a tube containing EDTA as anticoagulant. Cells were harvested by centrifugation for 10 min at 2,000 rpm and subsequently washed in PBS thrice. Cells were diluted to get a suspension of 1:10 in PBS. Test compounds were added to 1 ml of this suspension and incubated for 1 h at 37°C. Positive control was maintained with 1% Triton X-100. After incubation period, the tubes were centrifuged for 10 min at 2000 rpm. The supernatant was transferred to MTP, and the absorbance was measured spectroscopically at 450 nm. The percentage of haemolysis was calculated by the following formula (Ahmad et al., 2010)


$$\text{Percentage haemolysis}=\frac{\left(\text{Absorbance of treated sample}-\text{Absorbance of control sample}\right)}{\left(\text{Absorbance of positive control}-\text{Absorbance of control sample}\right)}\times 100$$

### (B) Effect of Individual and Synergistic Combinations of Phytocompounds on HBECs

HBECs were collected from healthy individuals with good oral hygiene by gently rubbing the inner mucosal surface of the cheeks with a sterile swab which was subsequently swirled and suspended in sterile PBS. HBEC suspension was used immediately after collection. The cell suspension from different individuals was pooled together and centrifuged at 3,000 rpm for 10 min. The supernatant was discarded, and the pellet was resuspended in PBS. Cells were counted using the Automated Cell Counter (Countess II FL, Invitrogen, United States) and adjusted to 5 × 10^5^ cells/ml. HBECs were then incubated with different concentrations of individual and combinations of piperine and thymol for 20 min at 37°C. Hydrogen peroxide was used as positive control; after incubation, cells were stained with crystal violet and observed under the microscope (Nikon Eclipse TsR2, Japan) (Priya and Pandian, 2020).

### Statistical Analysis

All the experiments were carried out in at least three biological replicates with at least two technical replicates, and values are presented as mean ± standard deviation (SD). To analyse the significant difference between the value of control and treated samples, one-way analysis of variance (ANOVA) and Duncan’s *post hoc* test were performed with the significant p-value of <0.05 by the SPSS statistical software package version 17.0 (Chicago, IL, United States).

## Results

### Minimum Biofilm Inhibitory Concentration (MBIC) of Piperine and Thymol Against *C. albicans*

**Figure 1:**
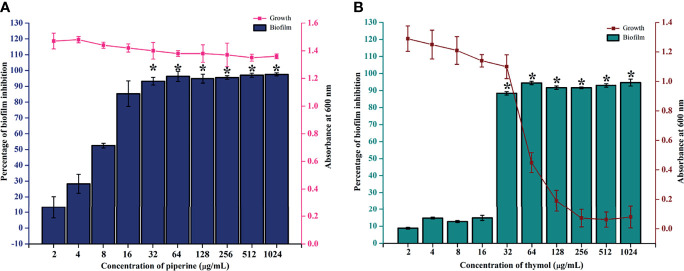
Determination of MBIC of piperine and thymol against *C. albicans.* **(A)** Piperine and **(B)** thymol at the 32-µg/ml concentration significantly reduced the biofilm formation without affecting the growth. Error bars represent standard deviations from the mean, and * indicates significance p < 0.05.

The biofilm inhibitory potential of piperine against *C. albicans* was reported in our previous study (Priya and Pandian, 2020). Piperine at 32 µg/ml inhibited the biofilm formation of the reference strain of *C. albicans* without affecting the viability (**Figure 1A**). From the present investigation, the antibiofilm potential of thymol was identified. At 32 µg/ml, thymol inhibited up to 90% of surface adherence and biofilm formation of the *C. albicans* reference strain without affecting the growth. A concentration higher than 32 µg/ml was found to be growth inhibitory in a dose-dependent manner (**Figure 1B**). Thus, 32 µg/ml was determined to be MBIC for both the phytochemicals piperine and thymol for the reference strain. MBIC was found to be slightly different for the clinical isolates. For CI-1, 128 µg/ml of piperine and thymol was determined as MBIC. For CI-2, 64 and 32 µg/ml of piperine and thymol, respectively, were identified as MBIC. The MBIC of piperine and thymol for CI-3 was identified as 32 µg/ml, whereas for CI-4, 32 and 64 µg/ml of piperine and thymol, respectively, was considered as MBIC (**Supplementary Figure 1**).

### Synergistic Antibiofilm Efficacy of Piperine and Thymol Against Biofilm of *C. albicans*

**Figure 2:**
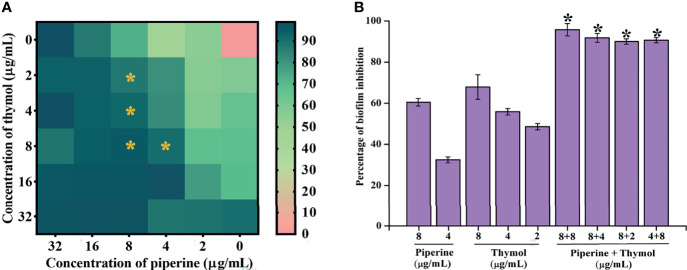
Synergistic antibiofilm efficacy of piperine and thymol against *C*. *albicans*. **(A)** Interaction of piperine and thymol in exhibiting antibiofilm efficacy was appraised through checkerboard analysis. Heatmap displays the percentage of biofilm inhibition by various combinations of piperine and thymol. Four different synergistic antibiofilm combinations of piperine and thymol were identified—8 + 8, 8 + 4, 8 + 2, and 4 + 8 µg/ml. **(B)** Comparative analysis of individual and combinatorial efficacy of piperine and thymol at synergistic combination concentrations against biofilm of *C*. *albicans*. Error bars represent standard deviations from the mean, and * indicates significance p < 0.05.

The interaction between piperine and thymol in exerting antibiofilm activity against *C. albicans* was analysed through checkerboard assay. The interaction was found to be synergistic at the following four different combinations of piperine and thymol in µg/ml: 8 + 8, 8 + 4, 8 + 2 and 4 + 8. The antibiofilm activity was found in other combinations. However, the FICI was found to be higher than 0.5 (**Table 2**). Thus, the aforementioned four combinations of piperine and thymol were determined to the synergistic combinations with antibiofilm activity against *C. albicans* (**Figure 2A**). When compared to the individual concentrations of piperine and thymol in synergistic combinations, the antibiofilm efficacy of the synergistic combinations was found to be higher (**Figure 2B**). The heatmap displaying the synergistic antibiofilm efficacy of piperine and thymol for clinical strains of *C. albicans* is provided in **Supplementary Figure 2**.

**Table 2:** FICI values of various combinations of piperine and thymol.

Concentration of thymol in µg/mL	Concentration of piperine in µg/mL					
	0	2	4	8	16	32
**0**	–	–	–	–	*	*
**2**	–	0.125	0.1875	0.3125*	0.5625*	1.0625*
**4**	–	0.1875	0.25	0.375*	0.625*	1.125*
**8**	–	0.3125	0.375*	0.5*	0.75*	1.25*
**16**	–	0.5625	0.625*	0.75*	1*	1.5*
**32**	*	1.0625*	1.125*	1.25*	1.5*	2*

The FIC indices of piperine and thymol combinations at different concentrations are tabulated in **Table 1**. The sign “*” indicates the presence of antibiofilm activity in individual and combination of drugs, and “-” indicates the absence of antibiofilm activity. The inhibition of biofilm was observed at most of the combinations, but the synergistic effect of piperine and thymol was observed at combinations of 8 + 8 µg/ml; 8 + 4 µg/ml; 8 + 2 µg/ml and 4 + 8 µg/ml of piperine and thymol with FIC indices of 0.5, 0.375, 0.3125 and 0.375 respectively (highlighted).

### Non-Growth Inhibitory Effect of Combinations of Piperine and Thymol

**Figure 3:**
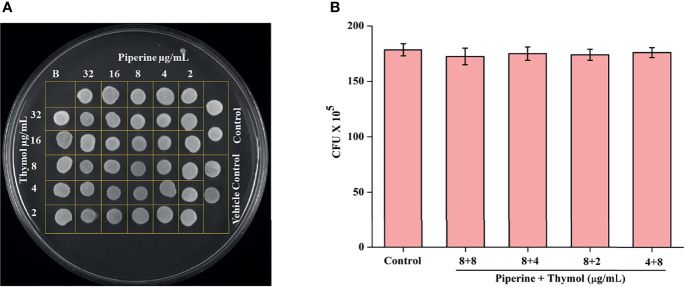
Non-growth inhibitory effect of synergistic combinations of piperine and thymol. **(A)** Spot assay displaying the growth of *C. albicans* treated with the individual and combined activity of piperine and thymol as compared to the control. **(B)** Bar graph representing the number of CFU by *C. albicans* grown in the absence and presence of identified synergistic combinations. No significant reduction in the growth was observed.

The effect of combinations of piperine and thymol on the growth of *C. albicans* was assessed through spot assay and CFU analysis. Individual and combinations of piperine and thymol were not found to exhibit any growth inhibitory effect on *C. albicans* as observed through the spot assay (**Figure 3A**). Control and synergistic combinations of piperine and thymol-treated *C. albicans* were spread plated in order to identify alterations in the CFU, if any. No significant change in CFU/mL was observed between the control and synergistic combinations treated, which validates that the combinatorial antibiofilm effect of piperine and thymol is not due to growth inhibition but rather to the phytochemical target only of the biofilm formation and virulence of the pathogen (**Figure 3B**).

### Piperine and Thymol Combination Influenced the Adhered Biofilm Cells of *C. albicans*

**Figure 4:**
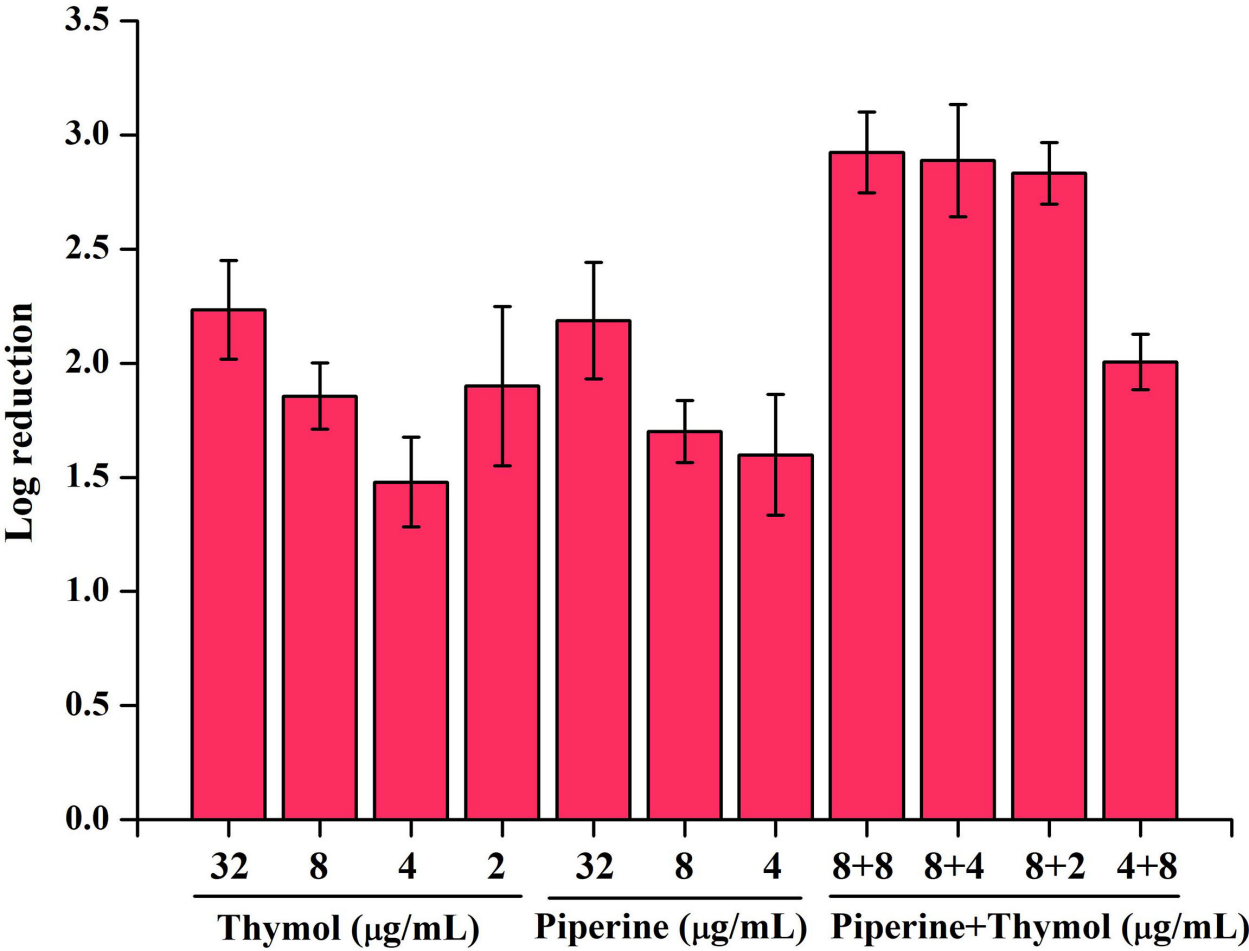
Effect of individual and synergistic combinations of piperine and thymol on biofilm cells of *C. albicans.* Adhered biofilm cells were found to be reduced above 2 log in MBIC of individual phytocompounds and their synergistic combinations.

Biofilm cells of *C. albicans* in the absence and presence of individual and synergistic combinations of piperine and thymol were subjected to CFU analysis. It is observed that the phytocompounds reduced the number of adhered biofilm cells drastically. A log reduction of more than 2 in biofilm cells was observed in treatment with phytocompounds at MBIC and synergistic combinations (**Figure 4**).

### Biofilm Architecture of *C. albicans* Under the Influence of Individual and Synergistic Combinations of Piperine and Thymol

**Figure 5:**
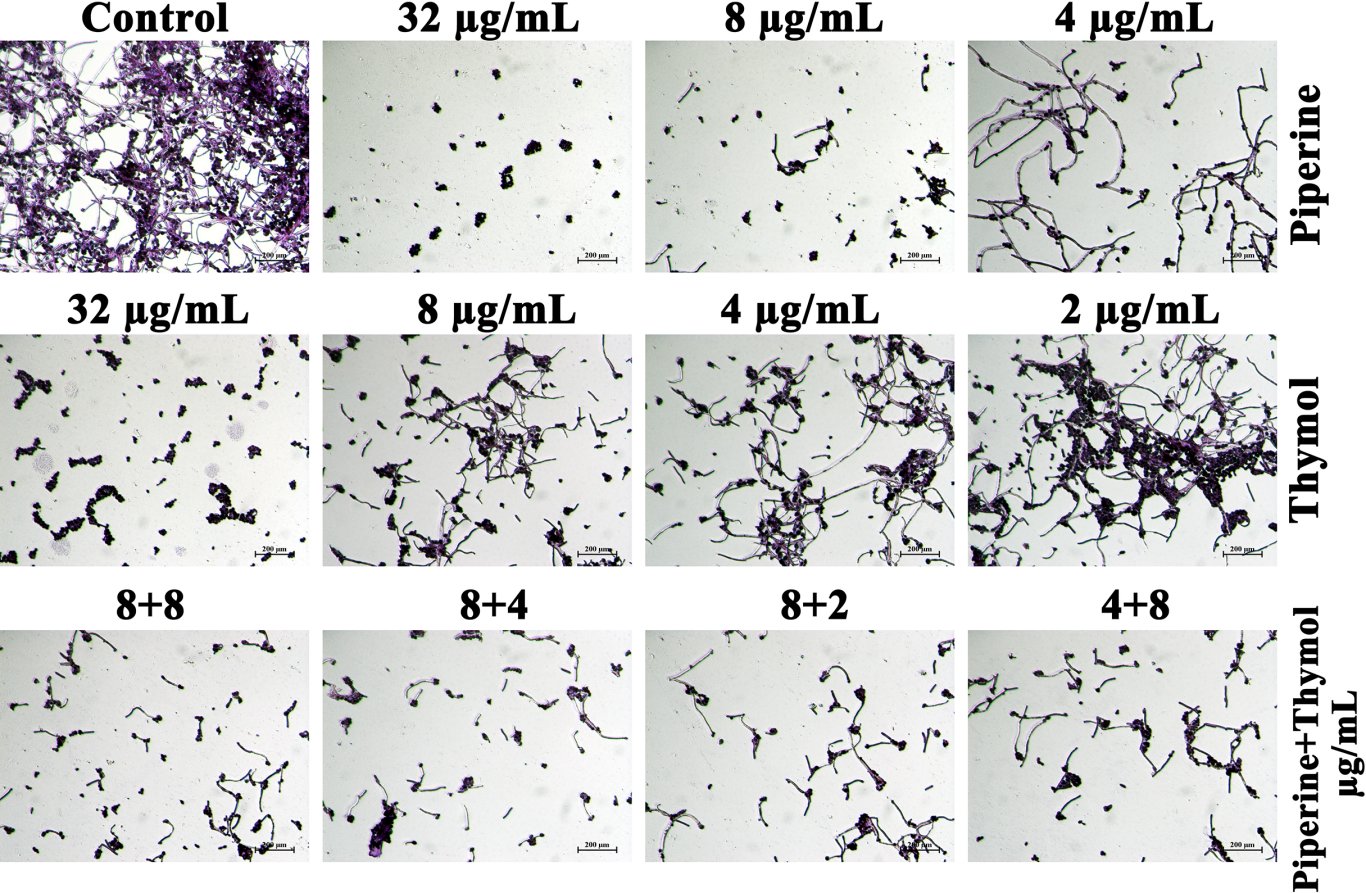
Microscopic visualisation of *C. albicans* biofilm formed under the influence of the synergistic effect of piperine and thymol. Biofilm of *C. albicans* without any treatment is encased with the conglomeration of yeast cells and filaments. Scarcely distributed yeast cells alone were found in the piperine and thymol-treated *C. albicans.* Few hyphal elongations were observed in treatment with sub-BIC of the phytochemicals. Synergistic combinations of piperine and thymol reduced the adherence of cells and filamentous morphology.

Adhesion of *C. albicans* cells to the glass surface was impaired by the influence of phytochemicals. Yeast cells entangled within the hyphal mesh were observed in the control whereas scarcely dispersed yeast cells without hyphal extension were observed in the *C. albicans* treated with MBIC of piperine and thymol. The individual effect of the concentrations in synergistic combinations was also analysed. Piperine at 8-µg/ml concentration resulted in few hyphal elongations. Although the number of adhered cells was lesser than the control, other individual concentrations in synergistic combinations of piperine and thymol resulted in hyphal morphogenesis, whereas the synergistic combinations of piperine and thymol significantly impeded the biofilm formation and hyphal elongation (**Figure 5**).

### Synergistic Antihyphal Efficacy of Piperine and Thymol

**Figure 6:**
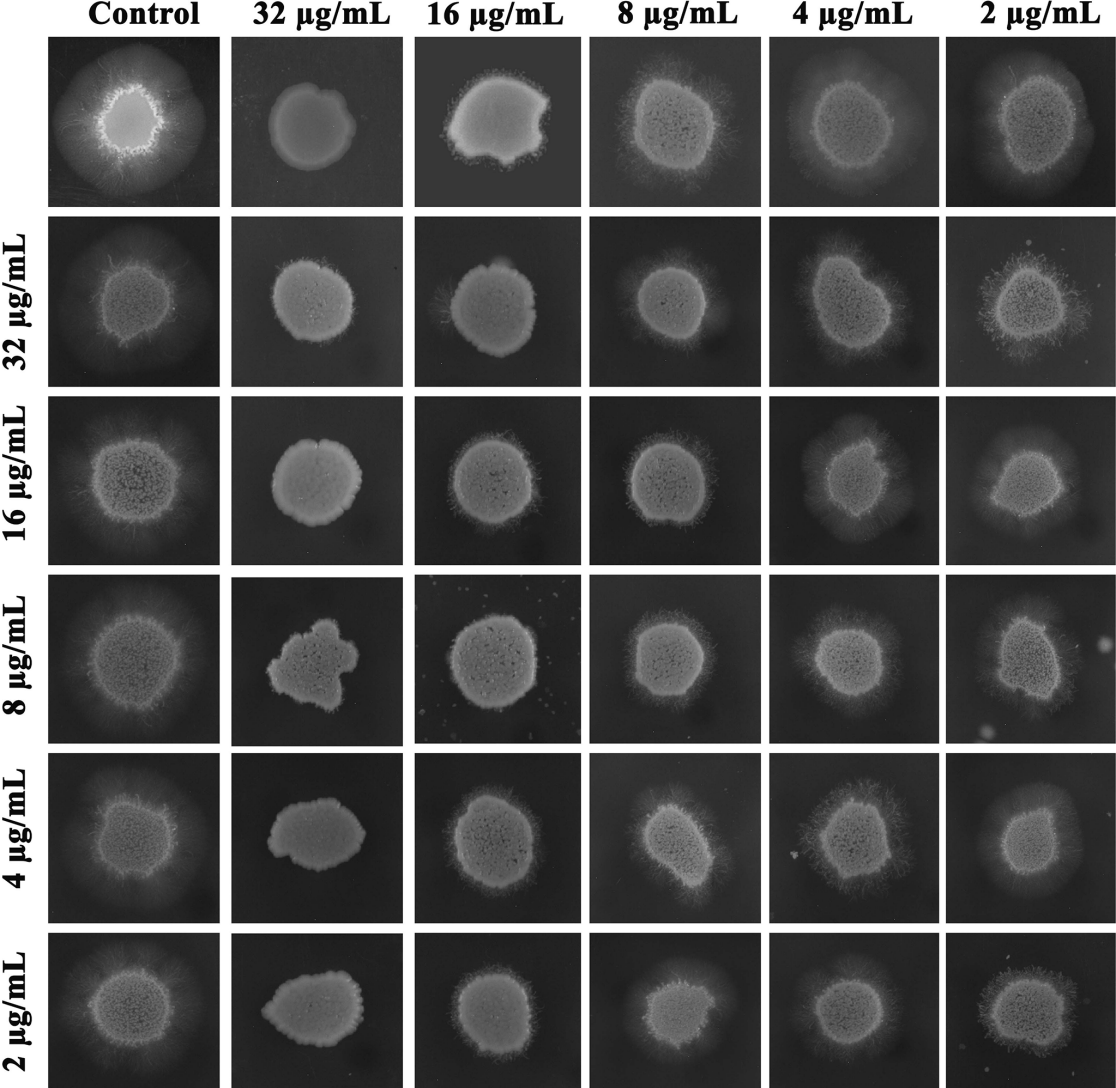
Antihyphal activity of individual and combination of piperine and thymol. Supplementation of FBS, a filament-inducing component, resulted in increased hyphal elongation around the cell surface of untreated *C. albicans*, whereas piperine at MBIC resulted in complete inhibition of hyphal form. Thymol at MBIC slightly inhibited the filamentation. At the 8 + 8-µg/ml concentration, synergistic antihyphal activity was observed with a FICI of 0.5.

Since the filamentous morphology of *C. albicans* was inhibited by the synergistic combinations of piperine and thymol in the microscopic observation, synergistic antihyphal activity was appraised. Compared to the individual MBIC of phytochemicals, a combination of piperine and thymol at a concentration of 8 + 8 µg/ml ensued in a synergistic antihyphal activity. The filamentous structure around the entire surface of the *C. albicans* colony was observed in control. Piperine at MBIC completely inhibited the hyphal growth. Synergistic antihyphal activity was observed at 8 + 8 µg/ml of piperine and thymol with an FIC index of 0.5. Certain other combinations were found to exhibit antihyphal activity, but the FICI was greater than 0.5 (**Figure 6**).

### Impact of Piperine and Thymol on Phenotypic Switching Between Yeast and Hyphal Forms

**Figure 7:**
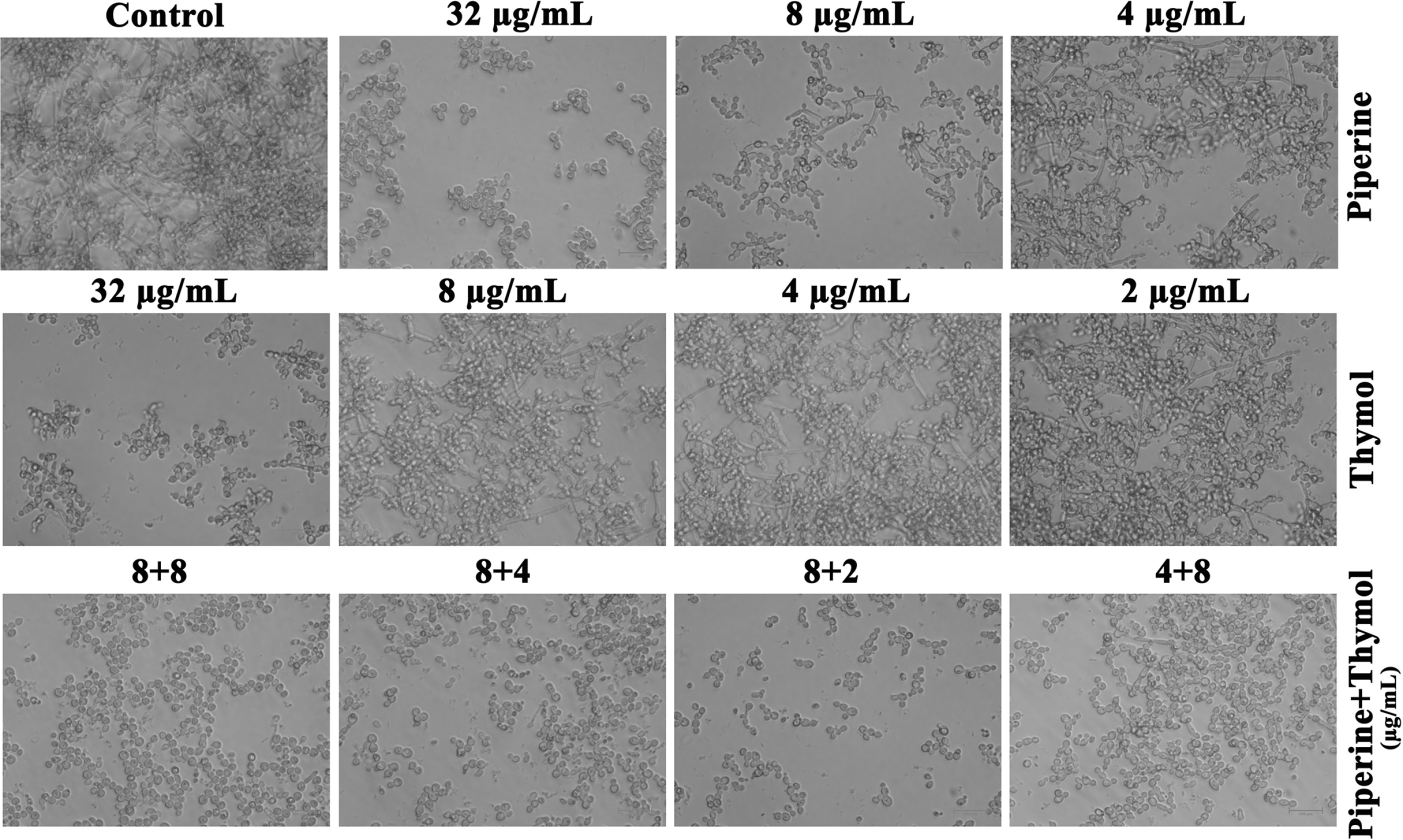
Impediment of phenotypic switch between yeast to hyphal form. Extension of hyphae was completely arrested by piperine and thymol at individual MBIC as well the identified synergistic combinations. Few filamentous extensions were observed in *C. albicans* treated with individual phytochemicals at the concentration present in synergistic combinations.

**Figure 8:**
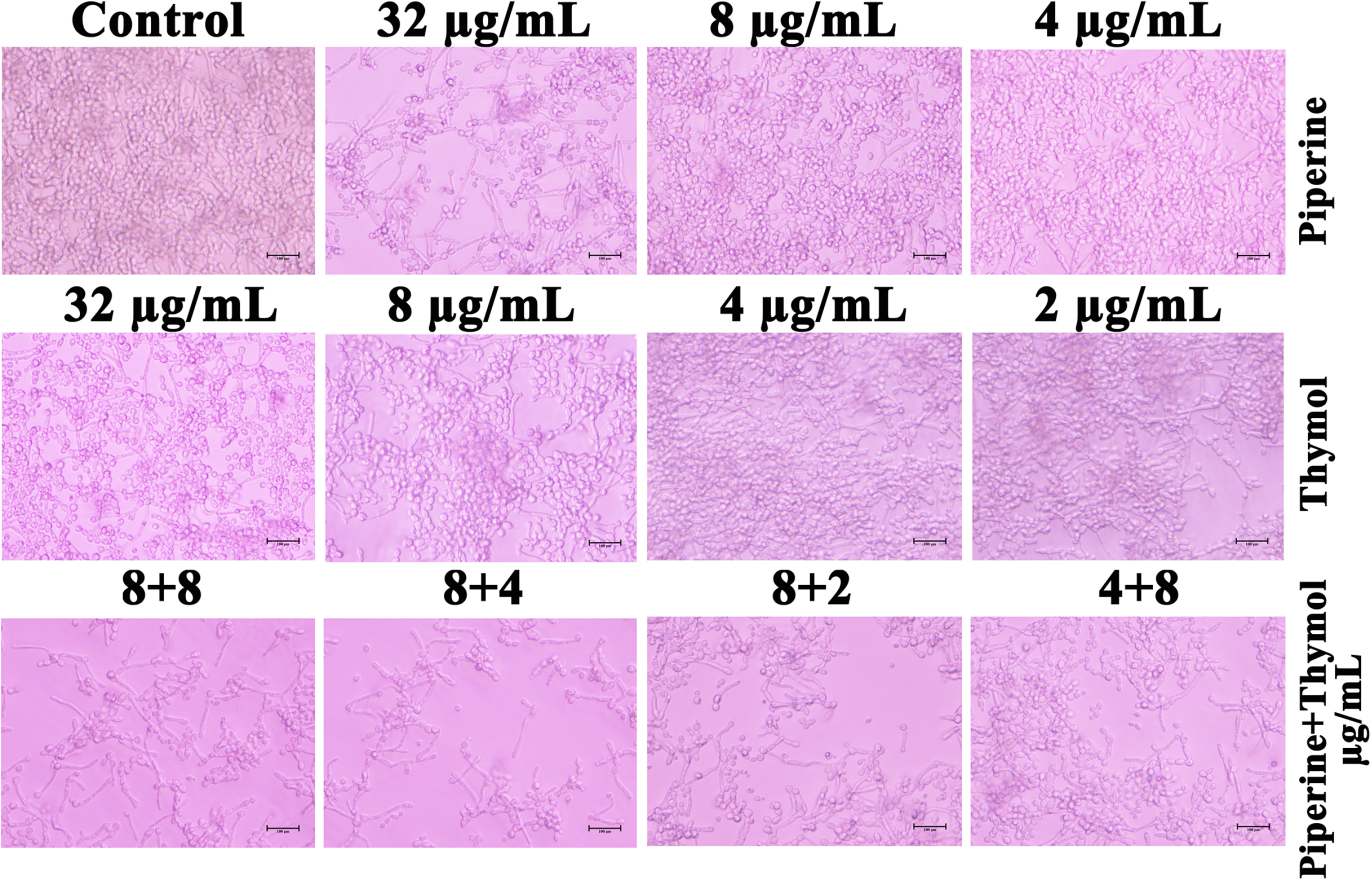
Reversion of hyphal to yeast morphology. Synergistic combinations of piperine and thymol exhibited proficiency in limiting and reverting the hyphal extension to yeast form.

Phenotypic switching in *C. albicans* is one of the major virulence determinants. Under hyphal-inducing conditions, the ability of the phytochemicals piperine and thymol in inhibiting the hyphal protrusion and relapsing the hyphal form to yeast morphology was assessed. FBS favourably enhanced the filamentous morphology in control, whereas the MBIC of piperine and thymol completely suppressed the filamentation. Similarly, only yeast cells were observed in the synergistic combinations (**Figure 7**). Contrarily, synergistic combinations of piperine and thymol limited and reversed the hyphal extension (**Figure 8**).

### Post-Antihyphal Effect

**Figure 9:**
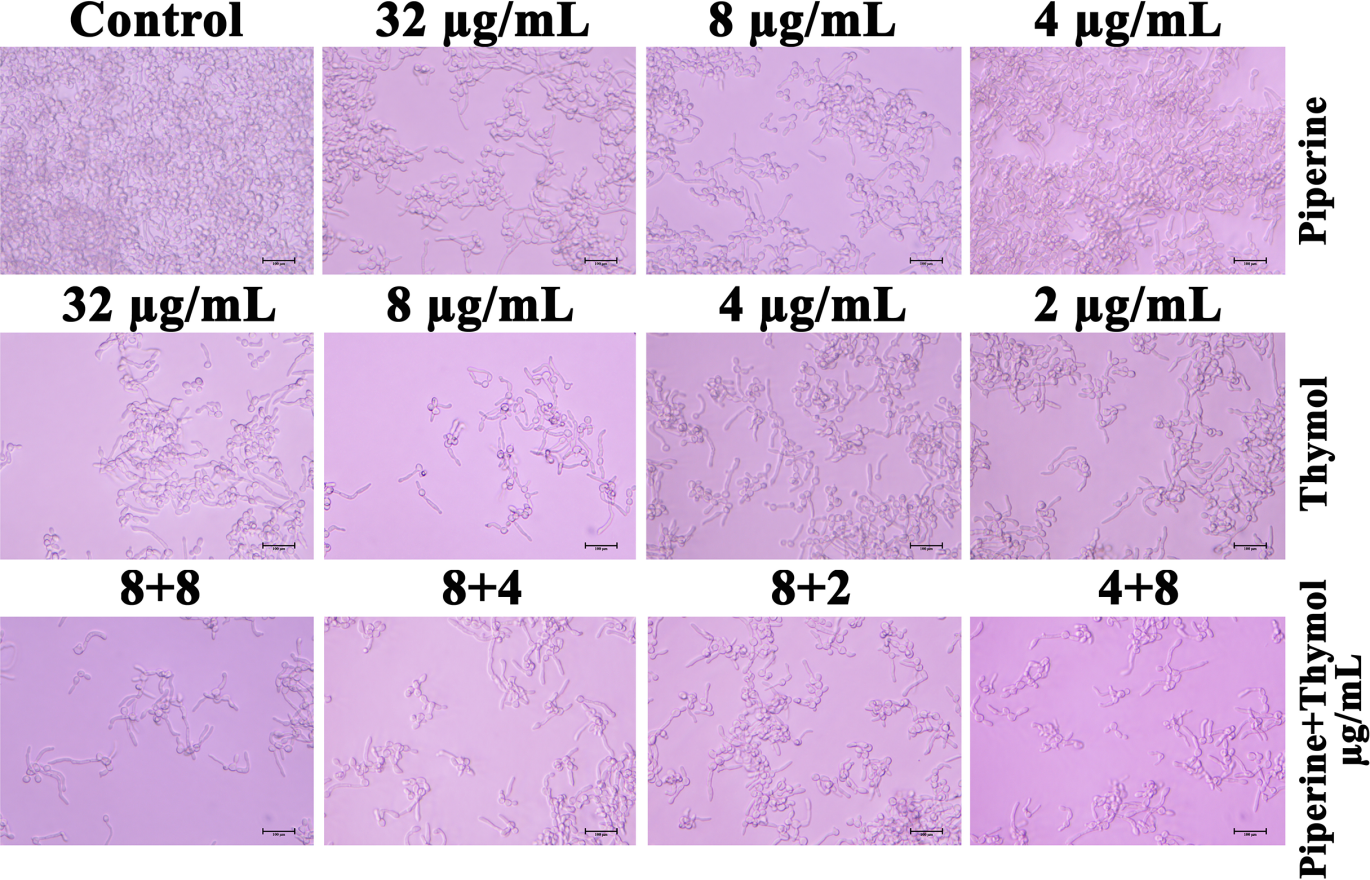
Short-term exposure of *C. albicans* cells with individual and synergistic combinations of piperine and thymol reduced/delayed the hyphal elongation process.

A brief exposure of *C. albicans* to individual and synergistic combinations of piperine and thymol limited the extension of filamentous morphology in hyphal inducing conditions. Also, yeast cell morphology was observed which could be due to the reversal of extended hyphae to yeast cells (**Figure 9**).

### Synergistic Combinations of Piperine and Thymol Enhanced the Susceptibility of *C. albicans* to Antifungals

The antifungal susceptibility of *C. albicans* treated with individual and synergistic combinations of piperine and thymol was found to be increased. The zone of inhibition in *C. albicans* exposed to synergistic combinations of piperine and thymol was comparatively equal or higher than the inhibition in *C. albicans* exposed to MBIC of individual phytochemicals (**Table 3**).

**Table 3:** Susceptibility of *C. albicans* to antifungals in the absence and presence of individual and synergistic combinations of piperine and thymol.

		Zone of inhibition in mm			
		Miconazole	Itraconazole	Ketoconazole	Nystatin
**Control**		26	16	26	17
**Piperine (µg/mL)**	**32**	27	17	28	18
	**8**	28	17	28	20
	**4**	30	18	29	18
**Thymol (µg/mL)**	**32**	29	20	30	19
	**8**	29	19	29	22
	**4**	28	18	28	19
	**2**	27	19	31	20
**Piperine + thymol (µg/mL)**	**8+8**	29	19	28	22
	**8+4**	29	21	29	21
	**8+2**	29	21	29	20
	**4+8**	29	18	26	17

### Regulation in the Expression of Candidate Virulence Genes

**Figure 10:**
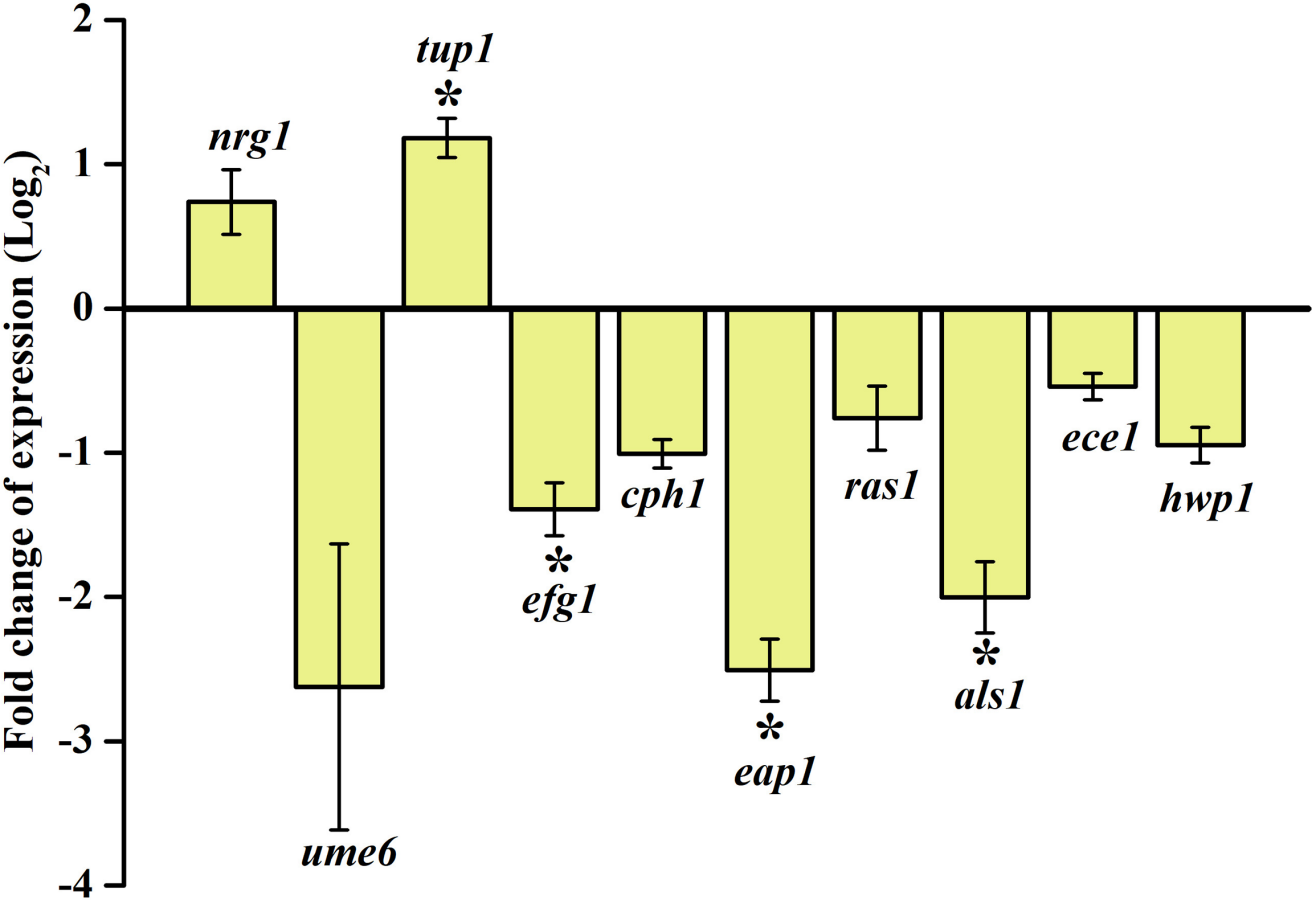
Dynamics in the expression of candidate genes under the influence of synergistic combinations of piperine and thymol. Negative and positive regulators of filamentation, *nrg 1*, *tup1*, and *ume6* are upregulated and downregulated, respectively. Other genes responsible for adhesion, biofilm formation, and filamentous growth are found to be downregulated.

Proficiency to inhibit biofilm formation and hyphal morphogenesis and to control the phenotypic switching between yeast and hyphal forms by the synergistic combination of piperine and thymol against *C. albicans* was validated through real-time PCR. Negative regulators of filamentation growth such as *nrg1* and *tup1* were upregulated whereas the transcriptional activator of filamentous growth, *ume6*, was downregulated. Filamentation and adhesion-related genes such as *efg1*, *cph1*, *eap1*, *ras1*, *als1* and *ece1* were found to be downregulated, which further corroborates the *in vitro* proficiency of piperine and thymol such as antibiofilm, antihyphal and control of phenotypic switch in *C. albicans* (**Figure 10**).

### Non-Toxic Nature of Piperine, Thymol and Their Combinations

**Figure 11:**
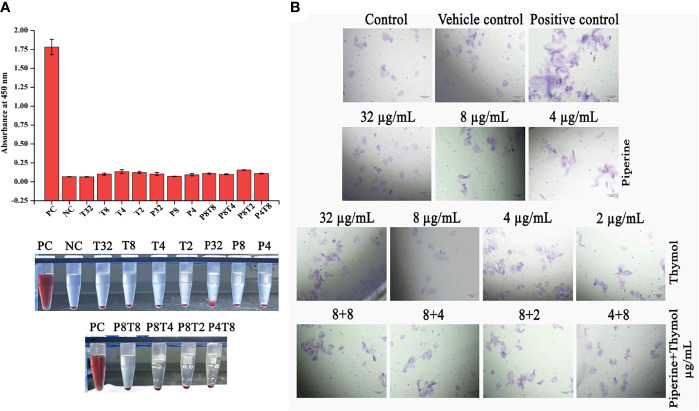
Non-toxic nature of phytocompounds. **(A)** Effect of phytocompounds and their synergistic combinations on human erythrocytes. No haemolytic activity was observed. As no haemolysis was observed, the graph was plotted with absorbance. Positive control Triton X-100 completely lysed the erythrocytes. **(B)** No toxic effect was observed on HBECs when treated with individual and combination of phytochemicals. Hydrogen peroxide (positive control) instigated morphological alterations in HBECs.

Phytocompounds piperine, thymol and their synergistic combinations were subjected to toxicity analysis on human red blood cells and HBECs. No toxic effect was observed at the tested concentrations in both erythrocytes and HBECs. In erythrocytes, the positive control Triton X-100 completely lysed the red blood cells as the treatment with phytocompounds did not lyse any detectable number of erythrocytes (**Figure 11A**). Similarly, no morphological alterations were observed in HBECs treated with phytocompounds whereas positive control hydrogen peroxide instigated alterations in the normal morphology of the cells (**Figure 11B**). These results demonstrate that the phytocompounds alone or in combination did not instigate any adverse effects to human erythrocytes or HBECs and thus can be considered safe for human application.

## Discussion

*C. albicans* being a eukaryotic organism shares several cellular mechanisms with humans. Thus, the identification and development of novel antifungal drugs without any adverse secondary effects on the human beings are a major confrontation. Conventional antifungal drugs target mechanisms or the end product that differs between fungi and humans. One such is the ergosterol biosynthetic pathway (Ksiezopolska and Gabaldón, 2018). Nevertheless, the emergence of resistance strains, including those with multidrug resistance are constantly being reported in recent years (Pfaller et al., 2013; Pfaller et al., 2014; Lockhart et al., 2017; Krishnasamy et al., 2020). To combat these challenges, the search for alternatives to antibiotics has been the recent theme of research. Numerous approaches such as antibiofilm therapy, use of antimicrobial peptides and phage therapy are recently being experimented for the treatment of infectious diseases to combat the persistent biofilm-associated infections. Among the various strategies that followed, the combinatorial approach is an emerging trend (León-Buitimea et al., 2020). The combination of drugs offers numerous advantages over monotherapy which includes reduced dosage, synergistic response, decreased possibility of resistance development and reduced toxicity (Lehár et al., 2009; Ahmed et al., 2014). To surmount these drawbacks, in the present investigation, the blended use of two emerging approaches such as antibiofilm therapy and the combinatorial strategy has been employed to control the oral candidiasis, which is a biofilm-mediated *C. albicans* infection.

Initially, the antibiofilm potential of piperine and thymol was evaluated using the standard crystal violet procedure. Alongside, the effect of these phytochemicals on the growth of *C. albicans* was also assessed as a potential antibiofilm agent should not affect the growth and vitality of the organism. The results displayed that piperine has no detrimental effects on the viability of *C. albicans* even at the highest tested concentration (1,024 µg/ml) whereas at 32-µg/ml concentration, more than 90% of *C. albicans* biofilm was found to be inhibited. Thymol was found to be slightly antifungal at higher concentrations. Hence, for thymol, 32 µg/ml was considered as BIC where more than 87% of biofilm inhibition was observed. Both the phytochemicals were found to be active against the biofilm of clinical isolates also but at slightly varying concentrations. The synergistic interaction between piperine and thymol was assessed through checkerboard assay. The interaction between piperine and thymol was found to exhibit synergistic activity. Four synergistic combinations were identified for the interaction between phytochemicals piperine and thymol, namely piperine + thymol combinations: 8 + 4 µg/ml; 8 + 4 µg/ml; 8 + 2 µg/ml and 4 + 8 µg/ml for the reference strain. Similarly, different combinations of piperine and thymol were found to exhibit synergistic antibiofilm activity against clinical isolates. Augmented antibiofilm activity was displayed by the synergistic combinations than the individual drugs in the concentration included in combinations. The possible growth inhibitory effect of the identified synergistic combinations was assessed through spot and CFU assay. No detrimental changes in the viability or significant changes in the number of cells were found between *C. albicans* grown in the absence and presence of synergistic combinations of piperine and thymol. Hence, it is evident that the identified synergistic combinations were found to exhibit antibiofilm activity without any effect on the growth of *C. albicans.* The *C. albicans* biofilm which is a conglomerate of yeast cells and hyphal forms along with the extracellular matrix is a major virulence attribute for persistent and recurrent infection. The effect of piperine and thymol combinations on the inhibition of biofilm formation was further validated through light microscopic analysis, and the results revealed that the synergistic combinations were effective in inhibiting the surface adherence of the cells as well as the hyphal elongation. Further, the combinatorial effect of piperine and thymol against the hyphal development, which is one of the major virulence factors of *C. albicans* required for the establishment of invasive infection, was assessed through solid agar assay. Various combinations of piperine and thymol were found to exhibit hyphal inhibitory activity, but only one synergistic combination with hyphal inhibitory activity was identified for piperine and thymol combinations. As the hyphal elongation was found to be suppressed, the effect of the synergistic combinations on the phenotypic switching ability of *C. albicans* was evaluated. Both yeast-to-hyphal transition and hyphal-to-yeast transition were significantly inhibited by the synergistic combinations which elucidate that the invading ability of *C. albicans* could be reduced when treated with piperine and thymol. Furthermore, the limited duration exposure of both the phytochemicals exhibited an antihyphal effect.

The *in vitro* results were validated through gene expressional analysis and found to be comparable. *nrg1* and *tup1* are negative transcriptional regulators of filamentous growth (Braun and Johnson, 1997; Braun et al., 2001). The expression of *nrg1* and *tup1* was found to be upregulated under the influence of piperine and thymol. In contrast, *ume6*, a positive regulator of the filamentation morphology, was found to be significantly downregulated. *efg*1 is reported as one of the filamentous morphogenetic regulators in *C. albicans* (Leng et al., 2001). Similarly, *cph1* also acts as an activator of filamentous growth (Braun and Johnson, 2000). *ras1* regulates the switch between yeast and hyphal forms (David, 2013). Whereas *als* and *eap1* encode for adhesins that facilitate the adherence of *C. albicans* to polystyrene and epithelial surfaces, *ece1* encodes for the candidalysin that regulates various virulence factors such as biofilm formation, adhesion and filamentation (Engku Nasrullah Satiman et al., 2020). All these virulence regulators were found to be downregulated by the influence of piperine and thymol. No toxic effects were observed on erythrocytes or HBECs by individual or combined effect of piperine and thymol.

Altogether, these results suggest that piperine and thymol, two major bioactive components with diverse activities, can interact synergistically in exerting antibiofilm and antihyphal activity against *C. albicans.* Hence, a synergistic combination of piperine and thymol can be possibly used in the formulation of dentifrices that are specifically recommended for the treatment of oral candidiasis.

## Data Availability Statement

The original contributions presented in the study are included in the article/**Supplementary Material**. Further inquiries can be directed to the corresponding author.

## Ethics Statement

The studies involving human participants were reviewed and approved by the Institutional Ethical Committee, Alagappa University, Karaikudi (IEC Ref No. IEC/AU/2018/5). The patients/participants provided their written informed consent to participate in this study.

## Author Contributions

SKP and AP designed the study. AP and SN performed the experiments. AP analysed the data, prepared the figures and tables and wrote the manuscript. SKP revised the manuscript. All authors have read and approved the final version of the manuscript.

## Conflict of Interest

The authors declare that the research was conducted in the absence of any commercial or financial relationships that could be construed as a potential conflict of interest.

## Publisher’s Note

All claims expressed in this article are solely those of the authors and do not necessarily represent those of their affiliated organizations, or those of the publisher, the editors and the reviewers. Any product that may be evaluated in this article, or claim that may be made by its manufacturer, is not guaranteed or endorsed by the publisher.
