# Supplementary material for: Synergistic Interaction of Piperine and Thymol on Attenuation of the Biofilm Formation, Hyphal Morphogenesis and Phenotypic Switching in Candida albicans
Source: Front Cell Infect Microbiol. 2022 Jan 19;11:780545. doi: 10.3389/fcimb.2021.780545 (PMC8807486; doi:10.3389/fcimb.2021.780545)

Synergistic interaction of piperine and thymol on attenuation of the biofilm formation, hyphal morphogenesis and phenotypic switching in *Candida albicans.*

Arumugam Priya, Srinivasan Nivetha and Shunmugiah Karutha Pandian*

Department of Biotechnology, Alagappa University, Science Campus, Karaikudi 630003, Tamil Nadu, India.

*Address for correspondence: Shunmugiah Karutha Pandian: pandiansk@gmail.com; Fax: +91 4565 225202; Tel: +91 4565 225215

Supplementary Figures

Supplementary Figure 1 Determination of MBIC of piperine and thymol against clinical isolates of *C. albicans*. Piperine and thymol at 128 µg/mL concentration significantly reduced the biofilm formation of CI-1. For CI-2, 64 µg/mL and 32 µg/mL of piperine and thymol, respectively were identified as MBIC. For CI-3 MBIC of piperine and thymol was identified as 32 µg/mL. Whereas for CI-4, 32 and 64 µg/mL of piperine and thymol, respectively was determined as MBIC. Error bars represent standard deviations from the mean and * indicates significance $p < 0.05$.

Supplementary Figure 2 Synergistic antibiofilm efficacy of piperine and thymol against clinical isolates of *C. albicans*. * indicates the concentrations were synergistic antibiofilm activities were observed.

Supplementary Figure 1

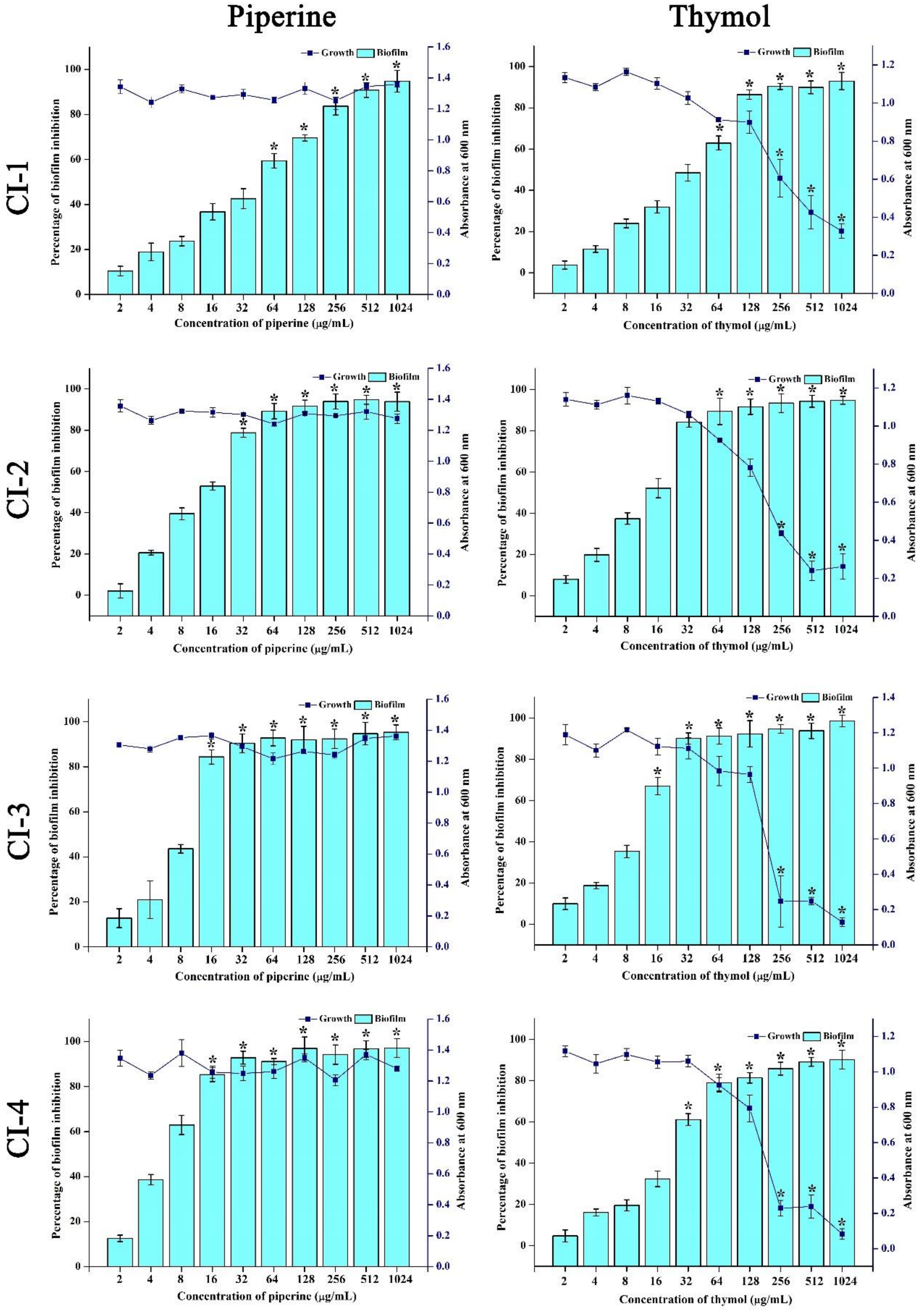

Supplementary Figure 2

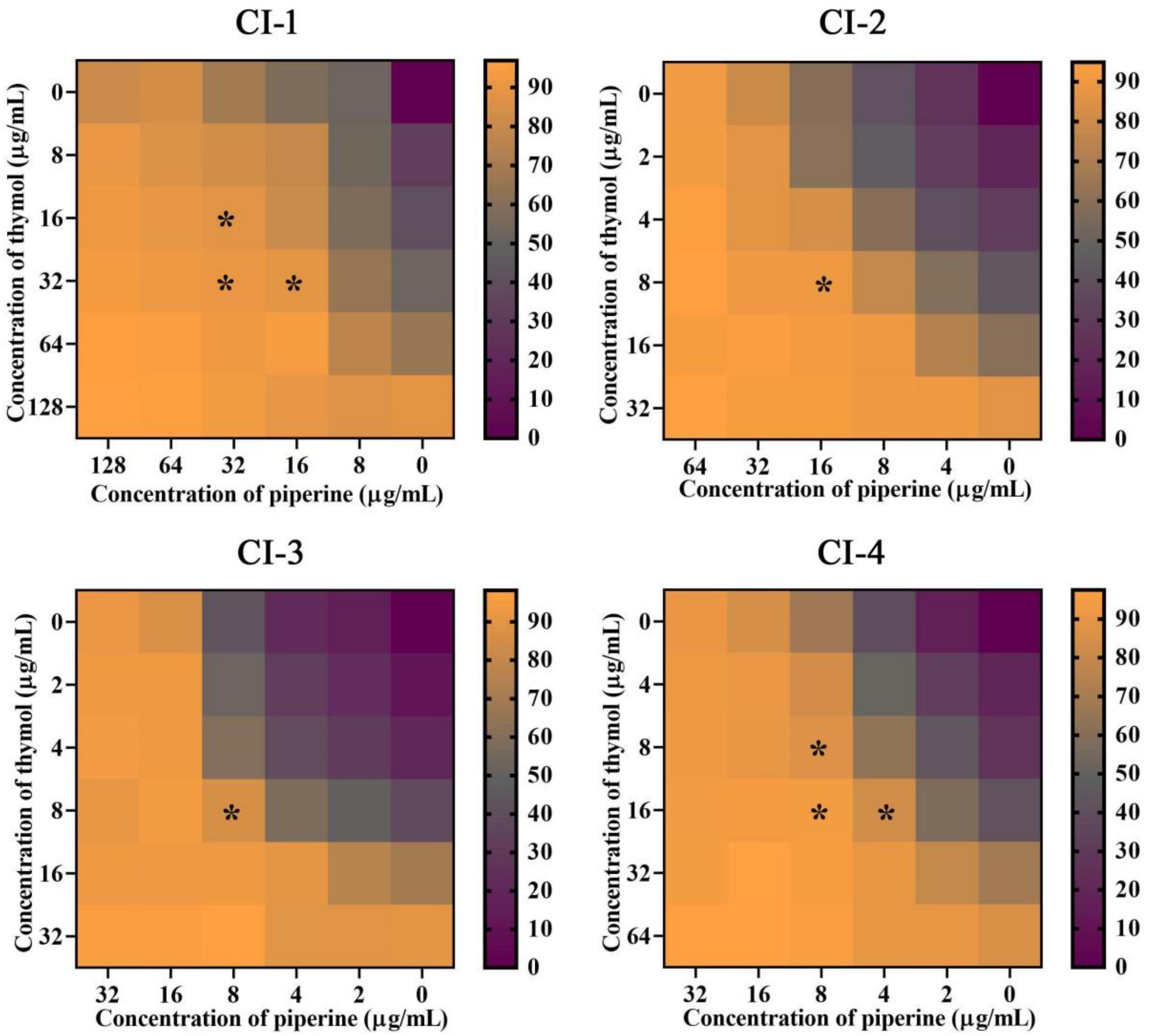

Supplement: Supplementary file 1 [file Image_1.pdf]
